# The Application of the Casting Process to Crown and Bridge Work

**Published:** 1911-05-15

**Authors:** Hart J. Goslee


### THE APPLICATION OF THE CASTING PROCESS TO CROWN AND BRIDGE WORK.

BY HART J. GOSLEE, B.S., D.D.S.,

The advent of the casting process has completely revolutionized all of our former methods; has made it possible to eliminate, or at least greatly diminish, many former deficiencies; has relegated a multitude of useless, impracticable, empirical methods to the memory of the past; has made it possible to follow a more or less scientific and systematic line of procedure, and to accomplish the combined requirements of *adaptation, strength and cosmetics* to the very highest degree, in a manner heretofore impossible.

In the application of the casting process the possibilities for obtaining almost perfect *adaptation* affords opportunity for the fitting of crowns, inlays and all forms of attachments to supporting roots, or teeth, with an almost wonderful degree of accuracy and certainty. It also insures a degree of uniform strength and of structural integrity not possible in former methods. These combined advantages make possible and practicable the use of less metal and more porcelain, thereby enabling us to more closely observe the very highest esthetic requirements.

This latter achievement is made possible in the average case, which constitutes a large percentage, by the use of all porcelain teeth instead of thin veneers, thus affording better form, better color and greater strength than could ever be obtained in the use of facings. Such were always known to be inherently weak, of poor form and doubtful color, owing to the necessary presence of a metal backing, and even when carefully protected by the display of gold, incisal ends and occlusal surfaces were frequently broken in soldering or in subsequent use.

Indeed, the frequency with which this occurred has been indicated of an element of structural weakness, and has stimulated a desire for some form of replaceable tooth which would adequately meet the demands.

Many such forms have been introduced to the profession, but up to the present most of them have been of the thin facing variety, and therefore more or less frail, and but little effort has been made to supply bicuspids and molars which would present occlusal surfaces of porcelain instead of gold.

The all-porcelain tooth, such as the Davis, Logan and Justi crowns, while originally designed for and used only in the restoration of a single tooth, where no better adaption than was to be obtained by grinding one surface to fit another was usually possible, and rarely attempted, have always been recognized as being the nearest approach to the ideal substitute for the natural teeth. Owing to the difficulty and uncertainty of obtaining the necessary closeness of adaptation to the supporting root, however, no great degree of permanency was insured, and their use has been limited to work usually regarded as being of a more or less temporary character.

If the esthetic advantages possessed by this type of tooth could be combined with those of close adaption and adequate strength, such as is now possible and if to these, combined, might be added the advantages of being cemented to the structure, and of being replaceable and more or less interchangeable, it would seem that our methods would indeed be revolutionized, and that our efforts would be a nearer approach to the ideal.

The attachment of porcelain teeth or facings to the metal structure by means of soldering, or even of casting is always fraught with uncertainty and danger, and in view of better methods, is technically wrong. It is undesirable because the porcelain is thus subjected to a degree of heat which must endanger its structural integrity and influence the preservation of its color. It is faulty because it is thus attached in a rigid and unyielding manner, and hence more likely to fracture under the stress of mastication; and it is objectionable because in the event of accident no opportunity for easy repair or replacement is afforded.

The elimination of such possibilities must necessarily constitute an improvement. The general use of replaceable teeth attached to the supporting structure by means of cementation. is reliable in proportion as the adaptation of the metal to the porcelain is accurate. It should be stronger because the porcelain is not subjected to heat. And it is better because by not being held so rigidly it is less likely to become fractured, by occlusion.

The greatest advantages, however, are to be obtained in a form of teeth which presents a mass of porcelain which is not weakened by metal pins, or other provisions for retention, therefore possessing a maximum of strength; it is of natural form, and more or less universally applicable and will require only a minimum of grinding for adaptation. The less the teeth are ground the easier they can be replaced.

All of these advantages, I believe, are to be found in a special tooth which has been devised by your essayist, which is being made by the Consolidated Dental Manufacturing Company.

They possess the strength and color of the Consolidated tooth bodies; are adapted to single crown work where any form of cast base is used, as well as to "dummies" for bridgework; affording a minimum display of gold and requiring but a minimum amount of grinding; and with a large variety of moulds—which are promised by the manufacturers—will be almost universally applicable.

These teeth are strongest where the greatest strength is required; they are provided with ample opportunity for a secure retention to the supporting base, or structure; and that the pins which are made for them—and which are to be furnished in iridio-platinum, clasp-metal (which will withstand 22 karat gold), and German-silver—will afford a secure means of attachment of the tooth to the casting.

In addition to this type of tooth, a *thin* replaceable facing for use in that limited class of cases involving the six or eight anterior teeth, where the conditions of occlusion or possible elongation of the opposing teeth demand a thin facing, has been suggested, and these with separable pins made of the same alloys are promised us in the near future.

These two general forms involving the same principles of attachment will insure universal application of the replaceable and interchangeable tooth. Hence I feel warranted in submitting that only this type of tooth should ever be used in single crown and "fixed" bridgework, if the best results and highest achievements are to be attained.

The supreme advantage offered by the use of replaceable teeth, however, is to be obtained by making duplicates. This requires but little time; is always a source of inestimable protection to the patient, and of unlimited relief and satisfaction to the dentist, and is a practice which should be observed by every one whose necessarily small fees do not make it prohibitive.

In adopting this practice the teeth shoulf be selected in pairs and when the original has been ground and the supporting casting made for it, the duplicate should then be ground to the same adjustment, which may be quickly and easily accomplished by painting the surface of the casting with black water-color paint. When the proper adjustment has been obtained and the assemblage of all of the parts (if it be bridgework, involving several teeth) has been completed the originals should then be cemented to place, and the duplicates numbered with pen and ink corresponding with the record kept, and then placed in some convenient form of small box, designated by the patient's name, and filed away for subsequent use in the event of accident.

In all instances, however, whether duplicates are made or not, the color number and mould number of each tooth should be recorded on the record sheet.

In additions to such improvements as have been achieved along these lines, the casting process has caused a multitude of empirical procedures heretofore in a common practice to become obsolete, and made more definite and better results possible.

As an evidence of the extent to which previous methods have been revolutionized, it is only necessary for me to briefly epitomize the present-day methods of crown and bridgework.

### SINGLE CROWNS.

For the ten anterior teeth, or all teeth within the range of vision, where porcelain is demanded, the all-porcelain replaceable crown, with cast base, and with or without a band, as the requirements may seem to indicate, is, next to a skillfully adapted "jacket" crown, the strongest and most artistic type of substitute for the natural tooth, and is, with vary rare exceptions, universally applicable. Indeed, it even possesses an advantage over the "jacket" crown in that immediate replacement is possible.

In the construction of this type of crown, however, the best results are usually obtained by first adapting a thin disk of pure gold to the end of the root, and attaching the dowel to it with a small bit of solder, and then casting directly to this disk. Otherwise, difficulty may be encountered in holding the dowel in its proper position, and in securing a close adaptation to both the basal end and periphery of the root.

For second and third molars, and even first molars, when the presence of gold is not objectionable, the cast gold crown is ideal. The peripheral adaptation, however, is best obtained by previously fitting a band to the root and casting directly to it. Where it is desirable to exaggerate the contour, this can perhaps be best accomplished by having the band fit snug round the periphery and trimming it to evenly approximate the end of the root, after which all of the contouring may be done in wax, and may be best done on a model.

Where an exaggerated contour is not required, however, the band may be made of 28 gauge, 22 karat gold, and fitted and contoured in the usual manner. When in position on the root, casting wax may then be moulded to the end of the root inside of the band, and followed by an imprint of the opposing teeth. When removed, the interior of the band should be filled with casting investment material and the occlusal surface then properly carved, after which it may be invested and cast, using the same grade of gold of which the band was made for the casting. If the band is thoroughly clean before investing and the gold to be cast is of good character and highly fused before casting, a good physical union will obtain.

Thus it will be observed that these two general types of crowns will meet the requirements of crown work in a very large majority of cases; leaving the "jacket" and the thin replaceable facing with cast backing for the special or exceptional cases.

### FIXED BRIDGEWORK.

In fixed bridgework which constitutes only an assemblage of "attachments" and intermediate "dummies," three general types of attachments will be found to meet the requirements in a large percentage of cases.

The porcelain replaceable crown with a cast base, and always with a narrow band for bridgework, as applied to the roots of anterior teeth; the inlay, always with some form of pin or post where the attachment is to be made to the crown of the tooth; and the gold crown for the molar teeth, where the use of an inlay is for any reason not indicated.

For "dummies" it will be found that three general types will also answer the requirements in practically all cases. The all-porcelain replaceable crown and bridge tooth with cast backing for all positions in the arch where the conditions of absorption and occlusion will permit. The backings for these teeth may be made separately, or in sections involving the number of "dummies" between the attachments, which should never exceed over three, or possibly four; and as a means of sustaining the proper relation of the post and of insuring a smoother surface adaptation to the porcelain than the ordinary casting investment materials afford, a thin backing of about 38 gauge pure gold should be burnished or swaged to the tooth, the post soldered to this, and the casting made directly upon it.

In the use of these teeth in cases where complete absorption has taken place, the most sanitary form of structure is usually to be obtained by moulding the wax to restore, somewhat, the lingual form of the tooth, and tapering down to a narrow saddle at the point of contact with the soft tissue. Such a type of construction where the adaptation is good will be found to be far more sanitary than the usual recesses, shelves and pockets so common in the ordinary methods of construction.

Next the thin replaceable facing for anterior teeth, where the occlusion and extent of overlapping demands thinness, and then the all-gold occlusal surface, or full-size "dummy" for posterior bridges, where cosmetics is not a factor, or where the extent of absorption or the elongation of opposing teeth precludes the use of porcelain. Such dummies may be made to conform to the requirements of occlusion, adaptations to gum, and contact with the attachments, and cast in one piece.

Thus the problem of "attachments" and of intermediate "dummies" for almost universal application is solved, all that remains being their proper assemblage.

Some are advocating the casting of all attachments and backings with an alloy of five per cent. of platinum in pure gold, and subsequently assembling the parts with pure gold, thus using no solder whatever. A good grade of 22 karat gold, or coin gold, may be used with equally good results, and the union of the parts can then be made with 22 karat solder. If there is absolute contact between the parts to be united, the procedure is somewhat facilitated, and the result all that is required.

### REMOVEABLE BRIDGEWORK.

The same general theme is also applicable to the construction of removable fixtures, in which class of work all the ingenuity and skill of the dentist are usually taxed to the fullest extent.

For this class of work, however, three general forms of anchorage to the supporting teeth or roots will be found to adequately meet the requirements of the average case: Clasps, the telescoping tube and split-post, and the manufactured attachments, such as the Roach and Morgan.

Wide clasps encompassing three angles of the tooth, provided with an occlusal rest, and made of heavy *rolled* clasp-metal alloy, probably afford the very best means of obtaining anchorage to the natural or artificial crowns of bicuspid and molar teeth. The telescoping tube and split-post attachment is particularly applicable to the roots of the six anterior teeth, and to projecting bars for extension bridges; while the Roach and Morgan forms of manufactured attachments will be found especially useful when used in connection with either porcelain or gold crowns, or inlays, on the cuspids and bicuspids.

When the type of attachment has been selected and adapted the casting process then offers a variety of opportunity, and many possibilities for the formation of the body of the denture. If a good model is obtained, and a good investment material be used, with coin gold as a basic metal, the process offers the same assurances of strength and accuracy of adaptation, as has been previously indicated.

In conclusion, my sense of justice prompts me to again call attention to the indebtedness of the profession to Dr. Taggart the master who has made such opportunities and improvements possible.

